# A Sensor Fault Detection Scheme as a Functional Safety Feature for DC-DC Converters

**DOI:** 10.3390/s21196516

**Published:** 2021-09-29

**Authors:** Simon Schmidt, Jens Oberrath, Paolo Mercorelli

**Affiliations:** 1 Institute of Product and Process Innovation (PPI), Leuphana University of Lueneburg, D-21339 Lueneburg, Germany; Simon_Schmidt1@gmx.net; 2 Department of Electrical Power Engineering, South Westphalia University of Applied Sciences, Luebecker Ring 2, D-59494 Soest, Germany; oberrath.jens@fh-swf.de

**Keywords:** DC-DC power converters, fault detection, Kalman filters, power system fault protection, safety

## Abstract

DC-DC converters are widely used in a large number of power conversion applications. As in many other systems, they are designed to automatically prevent dangerous failures or control them when they arise; this is called functional safety. Therefore, random hardware failures such as sensor faults have to be detected and handled properly. This proper handling means achieving or maintaining a safe state according to ISO 26262. However, to achieve or maintain a safe state, a fault has to be detected first. Sensor faults within DC-DC converters are generally detected with hardware-redundant sensors, despite all their drawbacks. Within this article, this redundancy is addressed using observer-based techniques utilizing Extended Kalman Filters (EKFs). Moreover, the paper proposes a fault detection and isolation scheme to guarantee functional safety. For this, a *cross-EKF structure* is implemented to work in *cross-parallel* to the real sensors and to replace the sensors in case of a fault. This ensures the continuity of the service in case of sensor faults. This idea is based on the concept of the virtual sensor which replaces the sensor in case of fault. Moreover, the concept of the virtual sensor is broader. In fact, if a system is observable, the observer offers a better performance than the sensor. In this context, this paper gives a contribution in this area. The effectiveness of this approach is tested with measurements on a buck converter prototype.

## 1. Introduction

Safety principles were initially considered in the military and nuclear areas and were then transferred to transport, process and control industries. Particular sector standards, dealing with safety critical points were defined afterwards. A large number of products and processes satisfy the standards of IEC 61508, for instance, automotive safety products, medical devices, sensors, actuators, diving equipment and process controllers. The international standard IEC 61508 (Functional Safety of Electrical/Electronic/Programmable Electronic Safety-related Systems) describes methods on how to apply, design, deploy and maintain safety-related systems. The adaption of IEC 61508 for Automotive Electric/Electronic Systems is ISO 26262, which defines functional safety as: “The absence of unreasonable risk due to hazards caused by malfunctioning behavior of electrical/electronic systems”. It further classifies malfunction of the electrical/electronic component into two types of failures:

- Systematic failures
- Random failures.

Systematic failures are induced during development, manufacturing, or maintenance (process issues) and can be addressed by safety management activities. Random failures are hardware failures due to aging processes or random defects. They are addressed by safety mechanisms that detect or control faults to achieve or maintain a safe state of the system. Some of these safety mechanisms are:

- Error correction code
- Hardware redundancy
- Built-in-self-test.

Detecting faults is one of the most challenging and important tasks in any field of application, see [1,2]. In the presence of strong noise, detecting faults becomes a difficult task. In [3], a new advancing coupled multi-stable stochastic resonance method, with two first-order multi-stable stochastic resonance systems, namely CMSR, is proposed to detect motor-bearing faults. Fault detection is also important in economy in which, very often, fault assumes the meaning of risk. In [4], a novel first-hitting time model is established to measure the relationship between the option reliability index and the value of risky assets. Detecting faults and isolating them using observer-based techniques has been applied to many technical fields. In this sense, a sensor fault detection scheme for induction motors was proposed in [5]. An aircraft engine fault diagnostic system based on Kalman Filters (KFs) was shown in [6]. Other examples for applied diagnosis systems are air conditioning [7], multilevel converters [8] and DC-DC converters [9].

To achieve functional safety, the term “hardware redundancy” often means redundant sensors, with all its drawbacks, including extra costs. In general, in the field of fault detection and safety, observers are used to establish a strategy to detect faults and errors. In [10], a Fuzzy observer based on Takagi–Sugeno (T-S) Fuzzy systems for sensor faults is proposed. In this work, sufficient conditions are obtained by determining the Fuzzy observer gains to detect sensor faults. However, in most publications, the observer used for fault detection is a KF-based scheme. The KF is one of the most important and widely used algorithms in the field of identification and observation for systems of any nature. After the seminal work proposed in [11], many different articles were proposed in the field of state observation which were summarized in surveys and books, see, for instance, the pioneer contributions in [12,13]. More application-oriented works are those in which discrete nonlinear systems are considered, as it is done in [14]. In this publication, it is shown that under certain conditions, the Extended Kalman Filter (EKF) is an exponential observer, i.e., the dynamics of the estimation error is exponentially stable. This is proven by the direct method of Lyapunov. An introduction to discrete KF and EKF can be found in [15]. More recently, in [16,17,18] and in [19], the author proposed different EKF structures for a valve control in an Otto motor application. Another emerging field in which EKFs are very often applied is the field state of charge estimation for batteries [20,21,22]. An up-to-date paper [23] shows further advancement of equivalent circuit model-based EKF. In the technical domain of synchronous drives, the KF has already found its way to many market-ready products. Nevertheless, there are still implementation pitfalls, which are addressed by [24]. Within the domain of power electronics, observers are used for a broad variety of tasks. The detection of grid fundamental and harmonic components for synchronization using a KF is described in [25]. In many cases, EKFs are used to estimate parameters. In [26], a new state observer dedicated to an online estimation of the model parameters is proposed. An example for parameter estimation of DC-DC converters using a KF can be found in [27]. Another approach to estimate parameters in this context is to use Adaptive infinite impulse response (IIR) filters, as [28] shows. In [29], a KF is utilized to estimate junction temperature of insulated gate bipolar transistor (IGBT) power modules.

**Figure 1:**
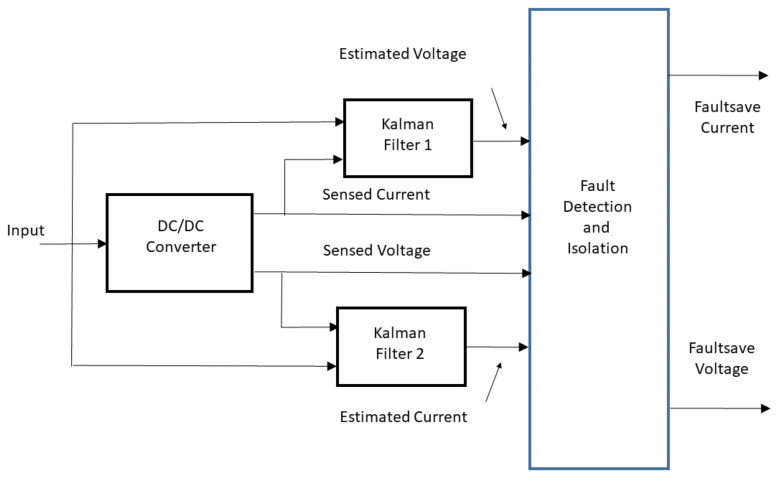
Conceptual scheme of Sensor Fault Detection.

**Figure 2:**
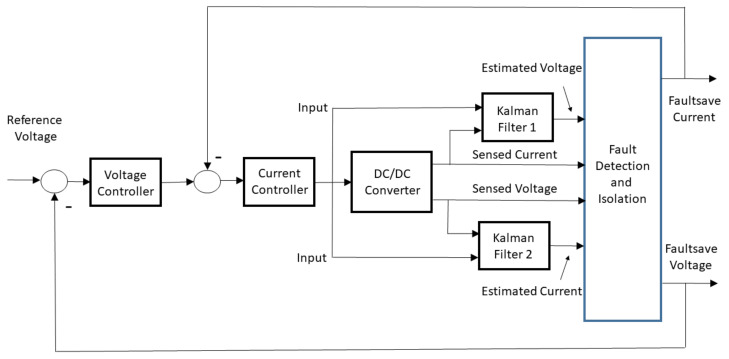
Conceptual scheme of Sensor Fault Detection with a possible control scheme.

For DC-DC converters, which need to be functionally safe by means of ISO 26262, redundant sensors are the standard safety mechanisms. In this context, this paper applies two *crossing Extended Kalman Filters* in order to estimate possible sensor faults as represented in Figure 1 and Figure 2. The idea is to cross the information obtained by the output state estimation (output voltage and output current) to detect the fault and to replace the faulty sensor.

This idea is based on the concept of the virtual sensor. A virtual sensor works parallel to the real sensor and replaces the real sensor in case of fault. Moreover, the concept of the virtual sensor is broader. In fact, if a system is observable, the observer offers a better performance than the sensor. The main contribution of this paper consists in the following:

- Building a DC-DC state space-averaged converter model;
- Developing an observer-based fault detection scheme, consisting of two crossing EKFs, see Figure 1 and Figure 2;
- Producing real measurements to show the effectiveness of the proposed technique.

The possible proposed scheme estimates the states of a DC-DC converter and detects the occurrence of sensor faults. Within the context of functional safety, the scheme is meant to work as a redundant sensoring structure implemented in software. The structure works in a *cross-parallel form* to the real sensors. This directly addresses random hardware failures by “achieving or maintaining a safe state” according to ISO 26262. More in depth, after a fault is detected, the other KFs provide the current signal and/or the voltage signal. In the case where no fault occurs, the signals *Faultsafe Current* and *Faultsafe Voltage* of Figure 1 and in Figure 2 are the sensor signals. If a fault is detected, the sensed signals are the estimated states generated by the cross-KF. In particular, in Figure 2, a possible scheme is shown in which we can see how this strategy can be utilized by a possible control scheme.

The paper is organized in the following way. Section 2 is devoted to the DC-DC Converter Model. Section 3 deals with the state estimation using augmented and extended Kalman filters. Section 4 shows the proposed method for fault detection and isolation. Experimental results and conclusions close the paper. In Appendix A, the Matlab code for fault detection scheme is shown explicitly for the user.

## 2. DC-DC Converter Model

**Figure 3:**
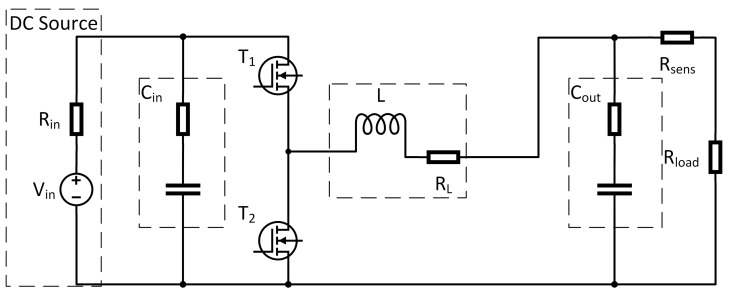
Equivalent circuit of a Buck Converter.

Many approaches are proposed to model DC-DC converters and one of the most popular review contributions can be found in [30] in which a clear picture on the general law and framework of the development of next-generation step-up DC-DC converters is presented. Real reviews and classifications of various step-up DC-DC converters based on their characteristics and voltage-boosting techniques are using concrete examples carried out. Another general overview on these devices can be found in [31], even though, more oriented on the dual-active-bridge isolated bidirectional DC-DC converter. One of these approaches is to describe the converter by means of a hybrid system model (see, e.g., [32]). Through this, a highly accurate model can be achieved in which the switching frequency directly indicates the sample time for the solver. Because of this, these kind of models lead to a high computational effort. The field of multi-harmonic modeling for DC-DC converters is recently reconsidered, for instance, in [33]. In [33], the proposed modeling technique is based on the large-signal averaged model of the PWM switch cell and on the Fourier series expansion. Since the aim of this paper is to detect sensor faults within a real time scenario, this model type is not reasonable. Another approach is to average over one switching period, which leads to a highly reduced dynamic of the model. There are several methods known from the literature which follow this general idea. In [34], most of these modeling techniques are covered. Within this reference, the methods are called “The Basic AC Modeling Approach”, “State-Space Averaging”, “Circuit Averaging” and “Averaged Switch Modeling”. In this article, the “State-Space Averaging” technique is chosen, because it leads to the most compact mathematical description of the system. A synchronous buck converter as shown in Figure 3 is considered in this contribution. It is assumed that the DC-DC converter only works in continuous conduction mode. This assumption means that the converter does not work under light or no-load conditions, which for most applications is a sufficient working condition. To apply averaging techniques, a first step is to separate the model by its switch positions.

**Figure 4:**
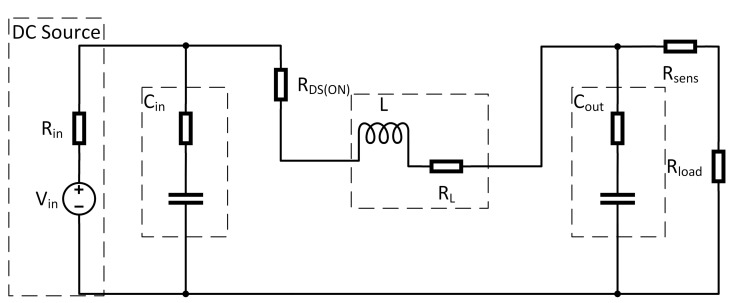
Equivalent circuit for $T=DT_{s}$.

Therefore, the circuit shown in Figure 3 is separated into two subintervals. The length of these intervals depends on the switching frequency $f_{s}=\frac{1}{T_{s}}$ and the duty cycle *D* of the pulse width modulated (PWM) switch control input. During the first subinterval, where switch $T_{1}$ is closed (switch $T_{2}$ open), the equivalent circuit reduces to the one shown in Figure 4. For this interval now, the differential equations in form of Equations (1) and (2) are derived:

(1) $$\begin{array}{cc} \frac{d}{dt}x\left(t\right)= & A_{1}x\left(t\right)+B_{1}u\left(t\right) \end{array}$$

(2) $$\begin{array}{cc} y(t)= & C_{1}x\left(t\right)+E_{1}u\left(t\right). \end{array}$$

**Figure 5:**
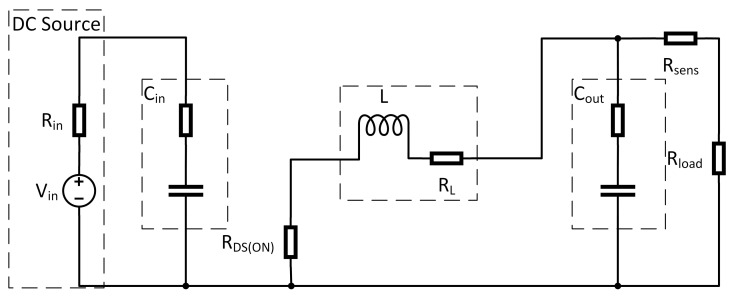
Equivalent circuit for $T=\left(1-D\right)T_{s}=T_{s}$.

For the second interval, where switch $T_{2}$ is closed ($T_{1}$ open), the remaining circuit is shown in Figure 5.

The next step is to derive differential Equations (3) and (4):

(3) $$\begin{array}{cc} \frac{d}{dt}x\left(t\right)= & A_{2}x\left(t\right)+B_{2}u\left(t\right) \end{array}$$

(4) $$\begin{array}{cc} y(t)= & C_{2}x\left(t\right)+E_{2}u\left(t\right). \end{array}$$

To obtain a description for the whole system, the subintervals are merged by:

(5) $$\begin{array}{ccc} A & = & DA_{1}+\left(1-D\right)A_{2}, \end{array}$$

(6) $$\begin{array}{ccc} B & = & DB_{1}+\left(1-D\right)B_{2}, \end{array}$$

(7) $$\begin{array}{ccc} C & = & DC_{1}+\left(1-D\right)C_{2}, \end{array}$$

(8) $$\begin{array}{ccc} E & = & DE_{1}+\left(1-D\right)E_{2}, \end{array}$$

where the A,B,C,E are averaged matrices for a full switching interval. Applying this method on the examined system in Figure 3, the averaged system matrices obtained are:

(9) $$\begin{array}{cc} A & =\left[\begin{array}{ccc} -\frac{R_{C_{in}}\;R_{C_{out}}+R_{C_{out}}\;R_{in}+2\;R_{C_{in}}\;R_{loss}+2\;R_{in}\;R_{loss}+2\;D\;R_{C_{in}}\;R_{in}}{2\;L\;\left(R_{C_{in}}+R_{in}\right)} & \frac{D\;R_{in}}{L\;\left(R_{C_{in}}+R_{in}\right)} & \frac{R_{C_{out}}-2\;R_{out}}{2\;L\;R_{out}}\\ -\frac{D\;R_{in}}{C_{in}\;\left(R_{C_{in}}+R_{in}\right)} & -\frac{1}{C_{in}\;\left(R_{C_{in}}+R_{in}\right)} & 0\\ \frac{1}{2\;C_{out}} & 0 & -\frac{1}{2\;C_{out}\;R_{out}} \end{array}\right], \end{array}$$

(10) $$\begin{array}{cc} B & =\left[\begin{array}{c} \frac{D\;R_{C_{in}}}{L\;\left(R_{C_{in}}+R_{in}\right)}\\ \frac{1}{C_{in}\;\left(R_{C_{in}}+R_{in}\right)}\\ 0 \end{array}\right],C=\left[\begin{array}{ccc} 1 & 0 & 0\\ 0 & 1 & 0\\ 0 & 0 & 1 \end{array}\right],E=\left[\begin{array}{c} 0 \end{array}\right],X=\left[\begin{array}{c} I_{L}\\ V_{C_{in}}\\ V_{C_{out}} \end{array}\right],U=\left[\begin{array}{c} V_{in} \end{array}\right], \end{array}$$

with

$$\begin{array}{ccc} R_{loss} & = & R_{on}+R_{L},\\ R_{out} & = & R_{sens}+R_{load}. \end{array}$$

These matrices fully describe the average system response. For the purpose of control design, often an AC small signal model is sufficient. This can be calculated with the following equations:

(11) $$\begin{array}{ccc} \frac{d\hat{x}\left(t\right)}{dt} & = & A\hat{x}\left(t\right)+B\hat{u}\left(t\right)\\ & + & \left[\left(A_{1}-A_{2}\right)X+\left(B_{1}-B_{2}\right)U\right]\hat{d}\left(t\right), \end{array}$$

(12) $$\begin{array}{ccc} \hat{y}\left(t\right) & = & C\hat{x}\left(t\right)+E\hat{u}\left(t\right)\\ & + & \left[\left(C_{1}-C_{2}\right)X+\left(E_{1}-E_{2}\right)U\right]\hat{d}\left(t\right), \end{array}$$

where the quantities $\hat{x}\left(t\right),\hat{u}\left(t\right),\hat{y}\left(t\right),\hat{d}\left(t\right)$ are small AC variations around the equilibrium solution. The steady state and output vectors are calculated by:

(13) $$\begin{array}{cc} X & =-A^{-1}BU,\\ Y & =(-CA^{-1}B+E)U, \end{array}$$

where X,U,Y,D are the DC equilibrium components:

- $X\;\hat{=}$ equilibrium state vector;
- $U\;\hat{=}$ equilibrium input vector;
- $Y\;\hat{=}$ equilibrium output vector;
- $D\;\hat{=}$ equilibrium duty cycle.

## 3. State Estimation Using Augmented Extended Kalman Filters

Since the model of the considered DC-DC converter was derived in the previous section, an observer based on the model can be designed. Due to the fact that the model described by Equations (11) and (12) is nonlinear in nature, the nonlinearity is taken into account in this step. For this task, there are several approaches known from the literature. This broad field of applications makes the KF and its extended version one of the most used estimation structures in the field of control systems. In this section, an EKF is implemented. Although the KF can be written as a single equation, it is more often described with two phases: the “Predict” and the “Update” phase. The “Predict” phase makes use of the previous estimation to predict the current state. Within the “Update” phase, the current prediction is combined with the current observation (measurement). This leads to an improved state estimation. These two phases can be summarized by the equations listed from Equation (14) to Equation (20).

*PREDICT PHASE*

Predicted (a priori) state estimate:

(14) $${\hat{x}}_{k|k-1}=f\left({\hat{x}}_{k-1|k-1},u_{k}\right).$$

Predicted (a priori) error covariance:

(15) $$P_{k|k-1}=F_{k}P_{k-1|k-1}F_{k}^{T}+Q_{k}.$$

*UPDATE PHASE*

Innovation (or measurement pre-fit) residual:

(16) $${\tilde{y}}_{k}=z_{k}-H_{k}{\hat{x}}_{k|k-1}.$$

Near-optimal Kalman gain:

(17) $$K_{k}=\frac{P_{k|k-1}H_{k}^{T}}{R_{k}+H_{k}P_{k|k-1}H_{k}^{T}}.$$

Updated (a posteriori) state estimate:

(18) $${\hat{x}}_{k|k}={\hat{x}}_{k|k-1}+K_{k}{\tilde{y}}_{k}.$$

Updated (a posteriori) estimate covariance:

(19) $$P_{k|k}=\left(I-K_{k}H_{k}\right)P_{k|k-1}.$$

Measurement post-fit residual:

(20) $${\tilde{y}}_{k|k}=z_{k}-H_{k}{\hat{x}}_{k|k}.$$

To set up the filter, the following matrices have to be determined:

- $F\;\hat{=}$ state transition matrix;
- $H\;\hat{=}$ measurement matrix;
- $R\;\hat{=}$ covariance matrix of measurement noise;
- $Q\;\hat{=}$ covariance matrix of process noise.

For the state estimation within the fault detection scheme, EKFs are used. It has, in general, the same structure as its regular version. The difference is that, the measurements or the state transition model, or both, are nonlinear. Due to this, the discrete time structure of the filter is utilized.

The state transition matrix *F* can be calculated by discretizing and calculating the Jacobian matrix of the model described in Section 2. Since the load $R_{load}$ of the DC-DC converter can change while the converter is working, it has to be taken into account. Therefore, another state is introduced by an augmented state variable $R_{out}$ through the following differential equation:

(21) $$\frac{d}{dt}R_{out}\left(t\right)\approx 0,$$

which states, in accordance with [15], the expression of a random constant which in the discrete form assumes the following expression: $R_{k}=R_{k-1}+w_{R_{k}}$, where $w_{R_{k}}$ represents the corresponding Gaussian white process noise associated with this equation. This state extends the model defined in Section 2 to four states, which need to be considered during further procedures. Next, the measurement matrix *H* needs to be determined. This matrix follows from the output equation, the system defined in Section 2 adding the augmented state. For practical implementations of the Kalman filter, the main difficulty is obtaining a good estimate for the noise covariance matrices $R_{k}$ and especially $Q_{k}$. $R_{k}$ contains the variance of the measurement. It can often be determined by knowing the standard deviation of the sensor system that is used in the system. Supposing a sensor has a standard deviation of σ, its *R* simply is $R=[\sigma^{2}]$, due to the fact that variance is the square of the standard deviation. Determining the matrix for the process noise $Q_{k}$ is a more challenging task. Procedures to adapt process noise covariance and measurements noise covariance matrices are known in the literature. In [35], different algorithms and methods are presented to calculate these two matrices using training data. The approach to guess this matrix is taken from [36], within this publication. Here, a set of discretely sampled points is used to parameterize this covariance matrix of the KF. In this way, the process noise covariance matrix is calculated with a sample of measurements. The process noise covariance is determined via calculations based on these measurements. By assuming that some measurements can be done on the system, the main idea of this approach is to utilize the sample mean of *N* measurements to obtain an estimate of this matrix. The sample mean vector can be defined as:

(22) $$\bar{X}=\frac{1}{N}\sum_{i=1}^{N}x_{i}.$$

Using these values as expected values, the deviation between observation and the sample mean can be computed as (see [37]):

(23) $$g={\hat{x}}_{k|k}-\bar{X}.$$

In matrix form, this is:

(24) $$G=\left[\begin{array}{cccc} {\hat{x}}_{1}-{\bar{X}}_{1} & {\hat{x}}_{1}-{\bar{X}}_{2} & \cdots & {\hat{x}}_{1}-{\bar{X}}_{n}\\ {\hat{x}}_{2}-{\bar{X}}_{1} & {\hat{x}}_{2}-{\bar{X}}_{2} & \cdots & {\hat{x}}_{2}-{\bar{X}}_{n}\\ \vdots & \vdots & \ddots & \vdots \\ {\hat{x}}_{n}-{\bar{X}}_{1} & {\hat{x}}_{n}-{\bar{X}}_{2} & \cdots & {\hat{x}}_{n}-{\bar{X}}_{n} \end{array}\right].$$

From this, the covariance matrix can be calculated as:

(25) $$Q=G\cdot G^{T}=\left[\begin{array}{cccc} \sigma_{1}^{2} & \sigma_{12} & \cdots & \sigma_{1n}\\ \sigma_{21} & \sigma_{2}^{2} & \cdots & \sigma_{2n}\\ \vdots & \vdots & \ddots & \vdots \\ \sigma_{n1} & \sigma_{n2} & \cdots & \sigma_{n}^{2} \end{array}\right].$$

σ represents the standard deviation of the states. This means, in the considered case, the matrix describes the variance between estimated states and the mean of measurement sample on the main diagonal. The other entries are the standard deviations between estimated states and different state measurement samples. For the sake of brevity, the details for the calculations of all these matrices are omitted. However, all necessary calculations are described within this section and the observer is implemented.

## 4. Residual-Based Fault Detection and Isolation

Now that the model and observer have been derived in the previous sections, the next step is to detect and isolate sensor faults. Therefore, every sensor under observation is equipped with a KF. In the case being considered, the voltage sensor and the current sensor are measuring the output voltage and the output current. Because these measurements are not equal to the states defined in Section 2, the following equations are used to fit the states to the measurements:

(26) $$\begin{array}{ccc} I_{out} & = & I_{L}-C_{out}{\dot{V}}_{C_{out}}, \end{array}$$

(27) $$\begin{array}{ccc} V_{out} & = & V_{C_{out}}+R_{C_{out}}C_{out}{\dot{V}}_{C_{out}}. \end{array}$$

For one observer, the measurement is the current and the estimated states are the remaining ones including the augmented state $R_{out}$. The other observer is provided by the voltage sensor data as measurements and estimates of the other states.

**Figure 6:**
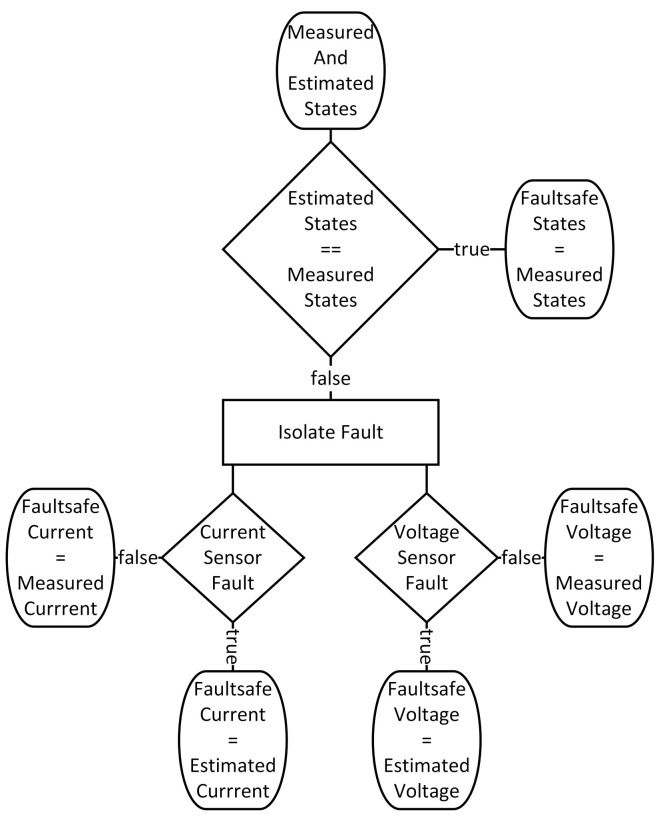
General process of sensor fault detection and isolation.

To detect and isolate sensor faults, measured and estimated states are compared with each other. If the deviation exceeds a limit, a first a fault is detected. After this recognition, the fault is isolated utilizing several logic operations (see Appendix A). Figure 6 shows the general workflow of how faults are detected and isolated.

The outputs of the fault detection scheme are called “Faultsafe States” from now on, in order to clearly separate them from measurements and estimated states. Firstly, the estimated and measured states are compared to each other. If they are equal (within a residual), the output (“Faultsafe State”) equals the measured states. If not, a fault is detected. In such a case, the fault has to be isolated, which means a distinction must be made between a current sensor fault and a voltage sensor fault. In case of a current sensor fault, the “Faultsafe Current” is set to the estimated current and the “Faultsafe Voltage” remains as the measured voltage. The exact opposite is the case in the event of a voltage sensor fault, meaning that the “Faultsafe Voltage” is set to the estimated voltage and the “Faultsafe Current” remains as the measured current. This guarantees that the proposed scheme in the event of a single sensor fault provides correct state signals for a controller. For better traceability and to gain a deeper insight, the program code of this procedure is provided in Appendix A.

## 5. Experimental Results

**Figure 7:**
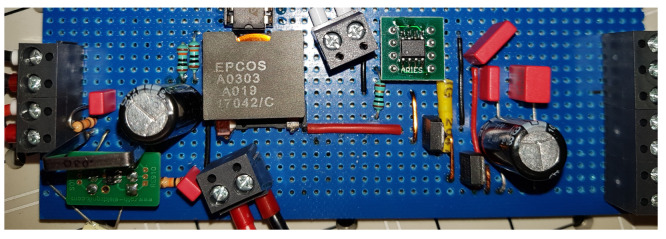
Buck converter prototype.

Since the general structure of the fault detection and isolation approach were derived in the previous sections, they have to be validated. For this purpose, a buck converter prototype was constructed (see Figure 7).

**Figure 8:**
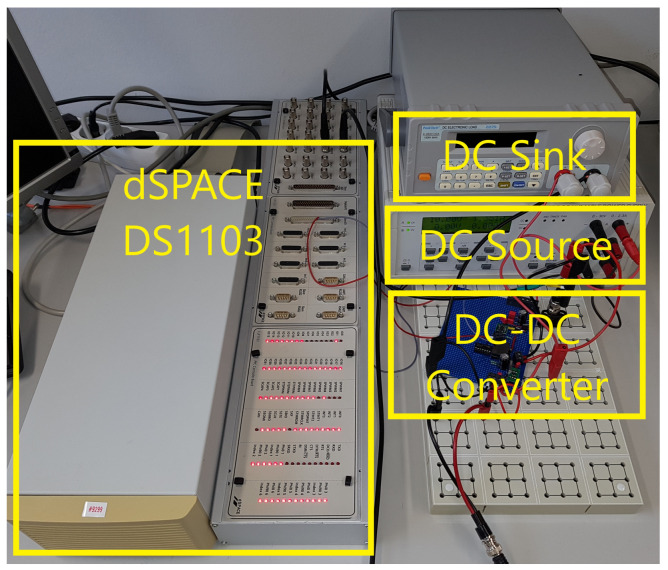
System for test purposes.

In order to generate PWM signals and do measurements, a “dSPACE DS1103” system was used. A “PeakTech 2275” electronic load functions as variable load. Furthermore, a “Grundig PN300” laboratory power supply was used to feed the system with power. The complete system installation is shown in Figure 8.

For the sake of completeness, the parameters of the DC-DC converter prototype used here are shown in Table 1.

**Figure 9:**
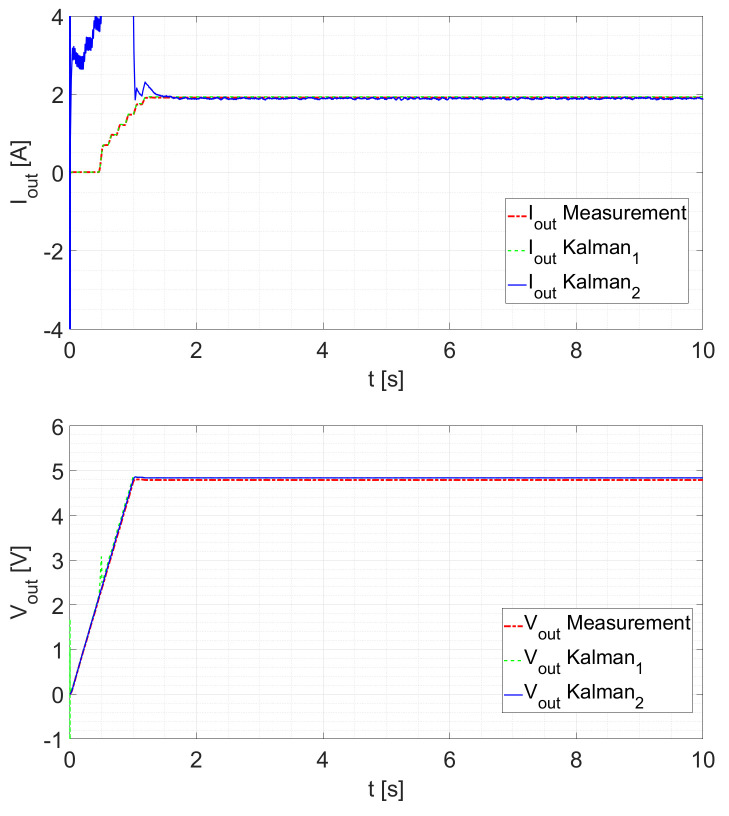
Ramp response for D=0 to D=0.5 at $R_{load}=2.5\;\Omega$.

The tests are done in a closed loop. As a first test, the Kalman filter response is tested by ramping up the duty cycle from D=0 to D=0.5 within one second. For $R_{load}=2.5\;\Omega$, the response is shown in Figure 9. It can be seen that the first Kalman filter follows the measured signal nearly exactly. The second filter follows the voltage measurement exactly, which is a consequence of being its measured state. The current estimation reaches the steady state in about 0.3s after *D* is constant (after the transient is completed).

**Figure 10:**
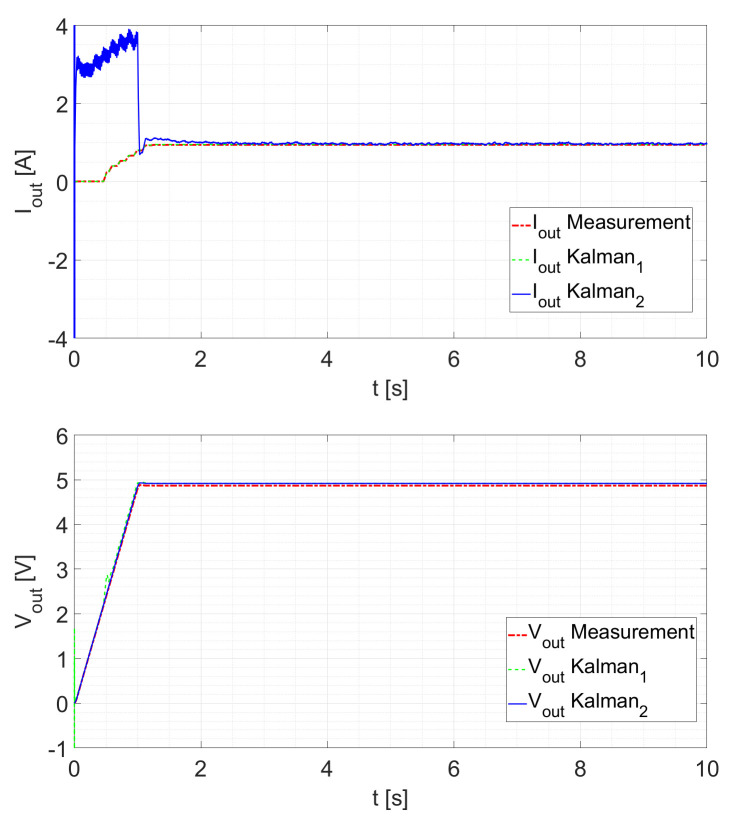
Ramp response for D=0 to D=0.5 at $R_{load}=5\;\Omega$.

As depicted in Figure 10 with $R_{load}=5\;\Omega$, both filters react in a similar manner. The $Kalman_{2}$ filter now attains its steady state in approximately 0.4s after the duty cycle is constant.

Comparing the different ramp-up tests with each other, it can be observed that for all tests, the voltage estimation is very accurate and fast. However, the current estimation for the second Kalman filter is less accurate until steady state is reached. Considering that the load is also an estimated state, this highly depends on its starting value. Other factors that can explain this behavior are:

- The starting values of the Kalman filters for the current estimation and especially the covariances;
- Current measurement becomes more inaccurate with smaller currents;
- Current sensor only works with a minimal current.

**Figure 11:**
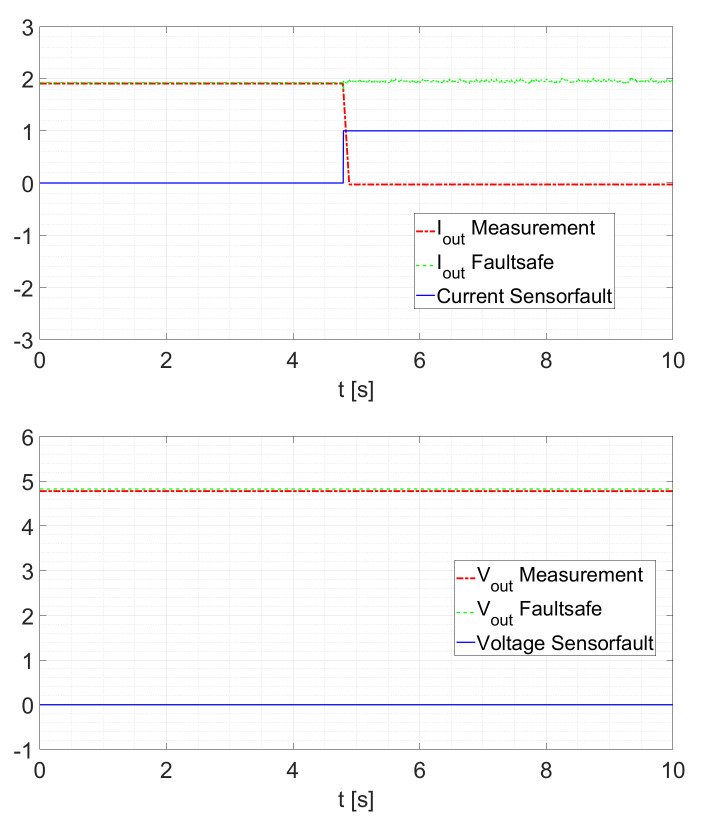
Current Sensor Fault at $R_{load}=2.5\;\Omega$.

**Figure 12:**
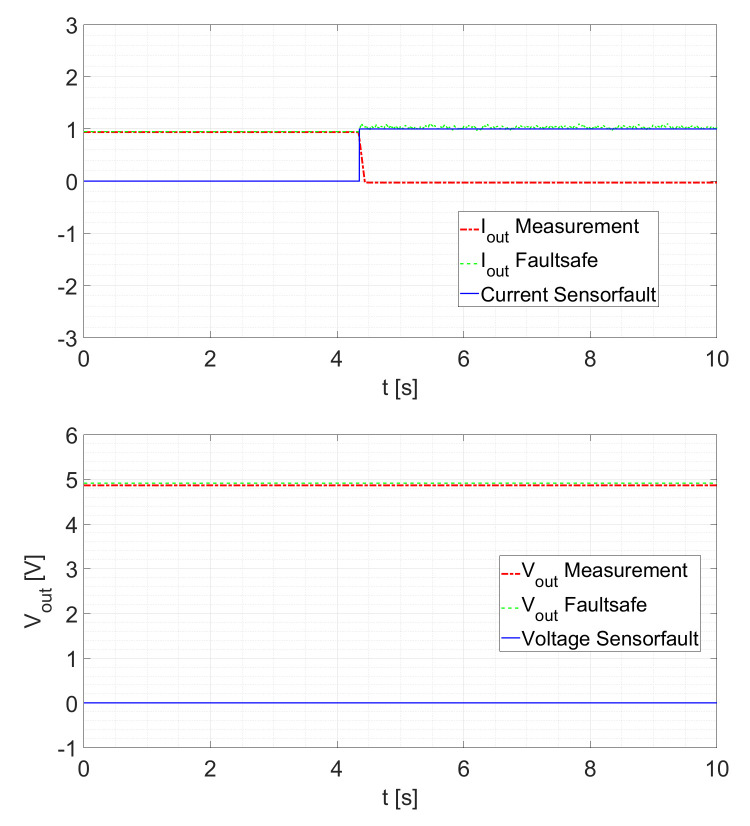
Current Sensor Fault at $R_{load}=5\;\Omega$.

Within the possible control scheme of Figure 2, the voltage loop is the outer loop. This means that the voltage controller provides the setpoint for the current controller. Therefore, a slower response of the current estimation will only slightly influence the controller response. A second test validates the sensor fault detection scheme. Here, the converter is operated at a constant duty cycle of D=0.5 and the sensor is disconnected at a random time. After a fault is detected, the other KF provides the current signal. In the case where no fault occurs, the signals $I_{out}Faultsafe$ and $V_{out}Faultsafe$ are the sensor signals. If a fault is detected, the signals are the estimated states generated by the cross-KF. As shown in Figure 11, a current sensor fault occurs at t≈4.8s. In this case, no interruption of the $I_{out}Faultsafe$ signal can be determined. For the voltage, the signal is still provided by the sensor. To recall, after a fault is detected, the other KFs provide the current signal and/or the voltage signal. In the case where no fault occurs, the signals *Faultsafe Current* and *Faultsafe Voltage* of Figure 1 and Figure 2 are the sensor signals. If a fault is detected, the signals are the estimated states generated by the cross-KF. In Figure 11 and Figure 12, it is possible to see how the KFs “replace” the faulted signal. In particular, in Figure 2, a possible scheme is shown in which we can see how this strategy can be utilized.

For a reduced electrical load of $R_{load}=5\;\Omega$ as displayed in Figure 12, the behavior remains similar.

**Figure 13:**
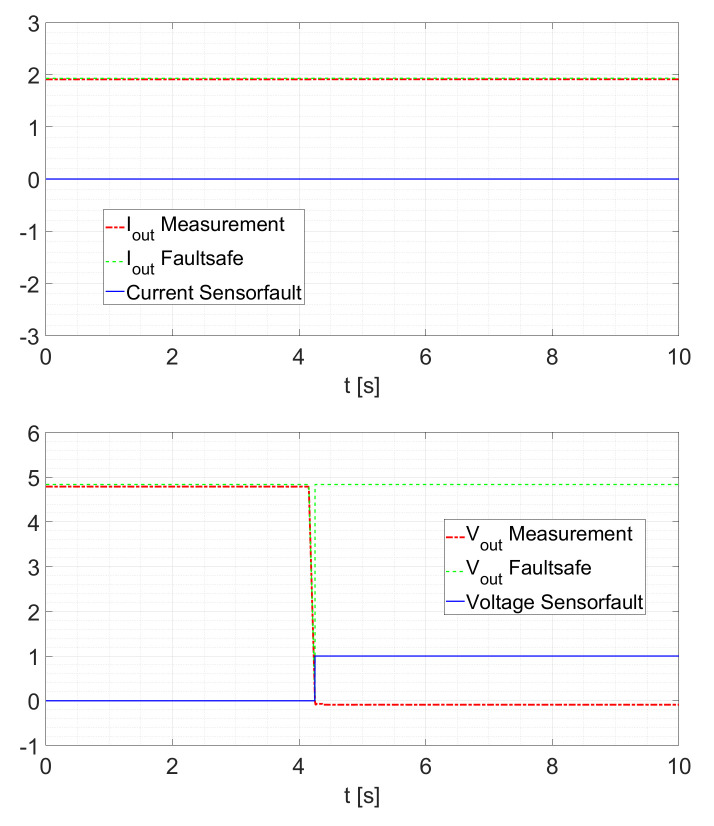
Voltage Sensor Fault at $R_{load}=2.5\;\Omega$.

**Figure 14:**
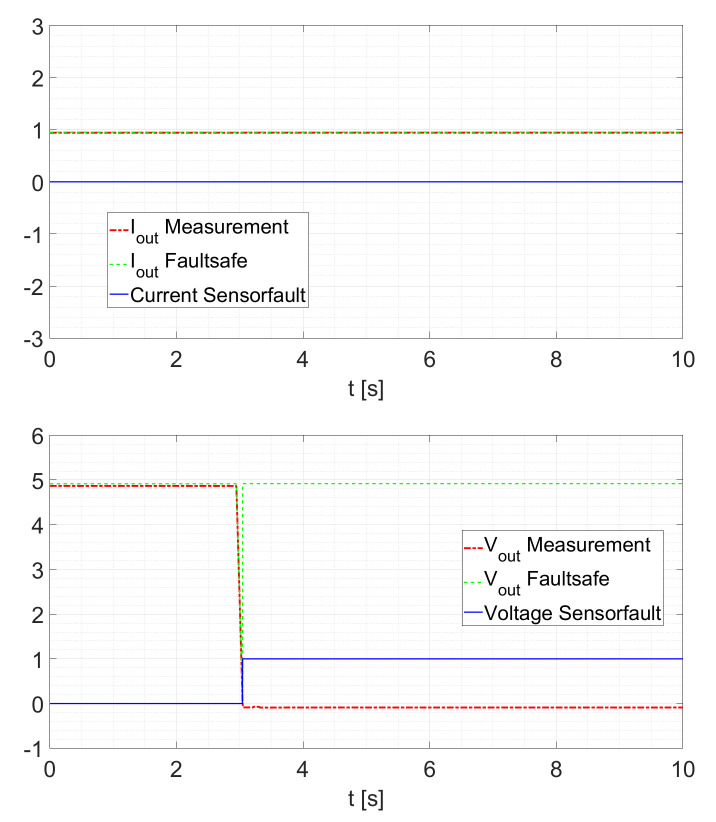
Voltage Sensor Fault at $R_{load}=5\;\Omega$.

**Figure 15:**
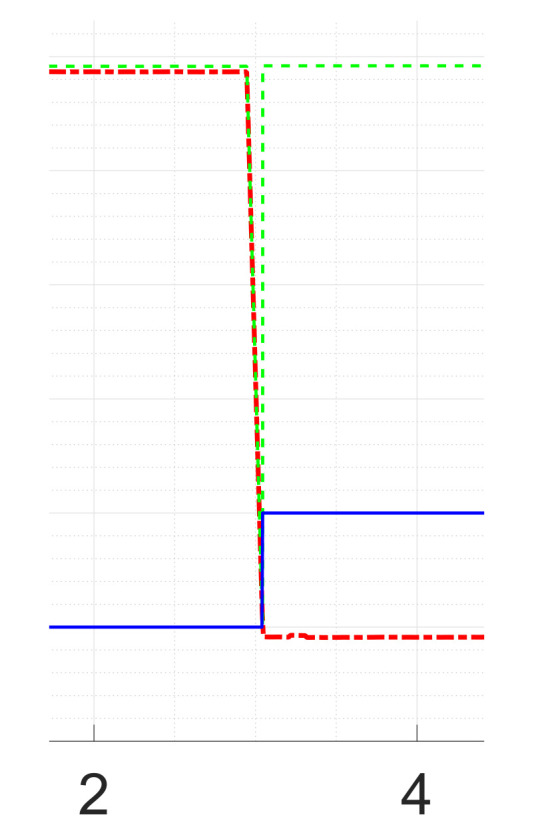
Particular of the voltage Sensor Fault at $R_{load}=5\;\Omega$.

Next, the voltage sensor fault detection is tested. The general test scenario remains the same. The sensor is randomly disconnected while the converter runs at a constant duty cycle of D=0.5. Doing so yields the response as shown in Figure 13. In fact, in Figure 13 and in Figure 14, it is possible to see how the KFs “replace” the faulted signal. It can be observed that, in such a situation, the fault detection takes some time to register the fault. From Figure 15, it is possible to see a particular of the voltage sensor fault. The transient takes around 100ms and the recognition of the faults is around 99% of the tested cases.

A further increase of $R_{load}$ to 5Ω leads to a response as it is depicted in Figure 14. The behavior remains similar to the first test.

From Figure 15, it is possible to see a particular of the voltage sensor fault. The transient takes around 100ms.

**Figure 16:**
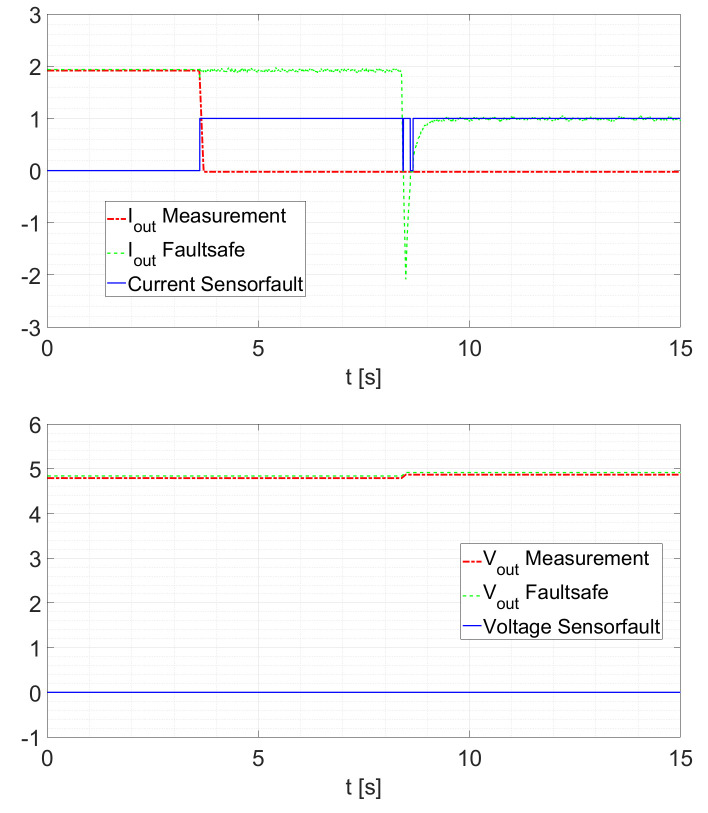
Current Sensor Fault at $R_{load}=2.5\;\Omega$ with load change to $R_{load}=5\;\Omega$.

**Figure 17:**
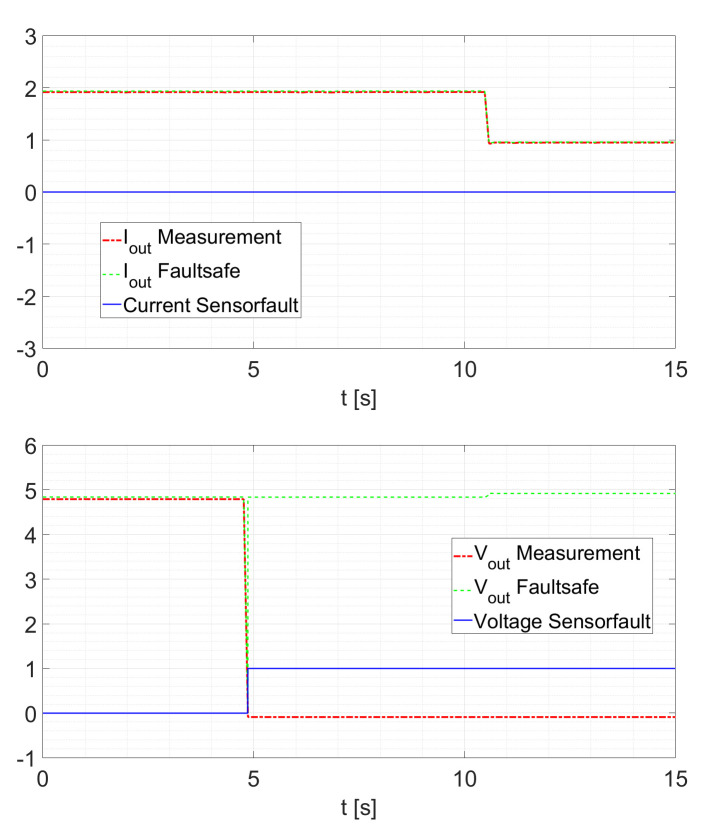
Voltage Sensor Fault at $R_{load}=2.5\;\Omega$ with load change to $R_{load}=5\;\Omega$.

The next test is performed in the following way. The sensor is disconnected at a random time and afterwards, the load is changed from $R_{load}=2.5\;\Omega$ to $R_{load}=5\;\Omega$. For a current sensor fault, the behavior is depicted in Figure 16. In addition, in Figure 16 and in Figure 17, it is possible to see how the KFs “replace” the faulted signal.

The same test with the voltage sensor produces the following response (Figure 17).

Under all tested scenarios, the fault detection detected the sensor fault. From a functional safety point of view, there are two possibilities to handle the detected faults:

- Switching off the converter by setting D=0;
- Continue operation with estimated signals.

From the definition of functional safety, “to achieve or maintain a safe state”, both handling options are in agreement with ISO 26262.

**Remark** **1.** *In the field of EKFs, typically, we are not able to prove the convergence of the estimation algorithm. The reason of that is rooted in the non-convexity of the optimization problem due to the nonlinear (switching) nature of the system. Nevertheless, the EKFs generate very good practical results in terms of performance, see [12]. In fact, if the filters are well tuned, the EKFs reach a good suboptimal performance almost always. Nevertheless, the tuning problem is in general a hard problem, in particular, if an adaptation of the process and of the measurement noise matrices is needed. In this specific case, due the stationarity of the problem, once the tuning process is realized, the EKFs offer a good result without any adaptation. The method used in this paper is taken from [36] and is sketched in Equations (22)–(25).*

## 6. Conclusions and Future Perspectives

The manuscript focuses on achieving functional safety within a DC-DC converter, without having hardware-redundant sensors. For this purpose, the converter under investigation is modeled. Since the proposed fault detection and isolation scheme are based on EKF, a brief description for this algorithm is given. Basing on this knowledge, a residual-based fault detection and isolation scheme are described. The proposed structure consists of a cross-combination of EKFs. This means that, the EKFs work in parallel with a sensor, but it is provided with the measurement of another one. Real measurements are included to show the effectiveness of the application. Future work includes the possibility to apply this algorithm to different architectures of DC-DC converters and to test also different control strategies such as, for instance, Sliding Mode Control. Due the structural non-convexity of the EKFs, in order to improve also the performance of the EKFs, a Particle Swarm Optimization Method can be possibly applied to improve the tuning of the EKFs.

## Figures and Tables

**Table 1:** Variables and parameters.

Symbol	Value	Description
$I_{L}\left(t\right)$	–	Current through the inductance (State variable)
$V_{C_{in}}\left(t\right)$	–	Input capacitor Voltage (State variable)
$V_{C_{out}}\left(t\right)$	–	Output capacitor Voltage (State variable)
$R_{out}\left(t\right)$	–	Load (Augmented state variable)
*D*	–	Duty cycle
$R_{L}$	0.075Ω	Winding resistance of inductance
$R_{DS(ON)}$	0.0395Ω	ON resistance of switch
$V_{in}$	10V	Input voltage
$R_{in}$	0.0001Ω	Resistance associated to the source input
$R_{C_{in}}$	0.095Ω	Resistance associated to the input capacitor
$R_{C_{out}}$	0.095Ω	Resistance associated to the output capacitor
$C_{in}$	180μF	Input capacitance
$C_{out}$	180μF	Output capacitance
$T_{s}$	$10^{-4}\;s$	Sample time

## Data Availability

Not applicable.

## Nomenclature

*A*	State-transition averaged matrix for a full switching interval
*B*	Input averaged matrix for a full switching interval
*C*	Output averaged matrix for a full switching interval
*E*	Input-output averaged matrix for a full switching interval
*D*	Duty cycle of the pulse width modulated (PWM) switch control input
$A_{1}$	State-transition matrix during the first subinterval $T=DT_{s}$
	where switch $T_{1}$ is closed and switch $T_{2}$ open,
	see Figure 3 and Figure 4
$B_{1}$	Input matrix during the first subinterval $T=DT_{s}$
	where switch $T_{1}$ is closed and switch $T_{2}$ open,
	see Figure 3 and Figure 4
$C_{1}$	Output matrix during the first subinterval $T=DT_{s}$
	where switch $T_{1}$ is closed and switch $T_{2}$ open,
	see Figure 3 and Figure 4

$E_{1}$	Input-Output matrix during the first subinterval $T=DT_{s}$
	where switch $T_{1}$ is closed and switch $T_{2}$ open,
	see Figure 3 and Figure 4
$A_{2}$	State-transition matrix during the second subinterval $T=\left(1-D\right)T_{s}$
	where switch $T_{1}$ is closed and switch $T_{2}$ open
	see Figure 3 and Figure 5
$B_{2}$	Input matrix during the second subinterval $T=\left(1-D\right)T_{s}$
	where switch $T_{1}$ is closed and switch $T_{2}$ open
	see Figure 3 and Figure 5
$C_{2}$	Output matrix during the second subinterval $T=\left(1-D\right)T_{s}$
	where switch $T_{1}$ is closed and switch $T_{2}$ open
	see Figure 3 and Figure 5
$E_{2}$	Input-Output matrix during the second subinterval $T=\left(1-D\right)T_{s}$
	where switch $T_{1}$ is closed and switch $T_{2}$ open
	see Figure 3 and Figure 5

## Appendix A. Matlab Code for Fault Detection Scheme

Within this code listed in Listing A1, variables ending in “M” are measurements. “K1” and “K2” denote an estimation by the first or the second Kalman filter. The “res” term denotes several residuals and “k” marks the actual time step.

**Listing A1.** Matlab commands for fault detection.
1 function [I_out, I_Fault, Vout, V_Fault]= ... 2 fcn (IoutM, IoutK1, IoutK2, VoutM, VoutK1, VoutK2) 4 I_Fault=0; 5 V_Fault=0; 6 ConI1=abs(IoutM-IoutK1) 7 ConI2=abs(IoutM-IoutK2) 8 ConV1=abs(VoutM-VoutK1) 9 ConV2=abs(VoutM-VoutK2) 10 CondI2=abs(IoutK2(k)-IoutK2(k-1)) 12 if ConI2>res1 && ConV2<res2 && ConI1<res3 13 if CondI2 < res4 14 I_Fault=1; 15 else 16 I_Fault=0; 17 end 18 else 19 I_Fault=0; 20 end 22 if ConV1>res5 && ConI1<res6 && ConV2>res7 23 V_Fault=1; 24 else 25 V_Fault=0; 26 end 28 if I_Fault==1 && V_Fault==0 29 Iout=IoutK2; 30 Vout=VoutM; 31 elseif V_Fault==1 32 Iout=IoutM; 33 Vout=VoutK1; 34 else 35 Iout=IoutM; 36 Vout=VoutM; 37 end

## Appendix B. Jacobian

Starting from the discrete state transition matrix, with the following calculations, the Jacobian matrix, implemented in the Extended Kalman Filter, can be derived:

(A1) $$A_{d}=I_{3\times 3}+T_{s}A$$

and, thus,

(A2) $$A_{d}=\left[\begin{array}{ccc} 1-T_{s}\frac{R_{C_{in}}\;R_{C_{out}}+R_{C_{out}}\;R_{in}+2\;R_{C_{in}}\;R_{loss}+2\;R_{in}\;R_{loss}+2\;D\;R_{C_{in}}\;R_{in}}{2\;L\;\left(R_{C_{in}}+R_{in}\right)} & T_{s}\frac{D\;R_{in}}{L\;\left(R_{C_{in}}+R_{in}\right)} & T_{s}\frac{R_{C_{out}}-2\;R_{out}}{2\;L\;R_{out}}\\ -T_{s}\frac{D\;R_{in}}{C_{in}\;\left(R_{C_{in}}+R_{in}\right)} & 1-T_{s}\frac{1}{C_{in}\;\left(R_{C_{in}}+R_{in}\right)} & 0\\ T_{s}\frac{1}{2\;C_{out}} & 0 & 1-T_{s}\frac{1}{2\;C_{out}\;R_{out}} \end{array}\right].$$

If the augmented state $R_{out}$ is considered, then the Jacobian matrix is as follows:

(A3) $$J_{d}=\left[\begin{array}{cccc} 1-T_{s}\frac{R_{C_{in}}\;R_{C_{out}}+R_{C_{out}}\;R_{in}+2\;R_{C_{in}}\;R_{loss}+2\;R_{in}\;R_{loss}+2\;D\;R_{C_{in}}\;R_{in}}{2\;L\;\left(R_{C_{in}}+R_{in}\right)} & T_{s}\frac{D\;R_{in}}{L\;\left(R_{C_{in}}+R_{in}\right)} & T_{s}\frac{R_{C_{out}}-2\;R_{out}}{2\;L\;R_{out}} & T_{s}\frac{-R_{C_{out}}}{2\;L\;R_{out}^{2}}\\ -T_{s}\frac{D\;R_{in}}{C_{in}\;\left(R_{C_{in}}+R_{in}\right)} & 1-T_{s}\frac{1}{C_{in}\;\left(R_{C_{in}}+R_{in}\right)} & 0 & 0\\ T_{s}\frac{1}{2\;C_{out}} & 0 & 1-T_{s}\frac{1}{2\;C_{out}\;R_{out}^{2}}\\ 0 & 0 & 0 & 1 \end{array}\right].$$

It is to recall that $T_{s}=10^{-4}$ which implies a sampling frequency of 10kHz.
